# 3D Urchin-Like CuO Modified W_18_O_49_ Nanostructures for Promoted Photocatalytic Hydrogen Evolution under Visible Light Irradiation

**DOI:** 10.3390/nano11010104

**Published:** 2021-01-04

**Authors:** Hongyu Ma, Yaqi Tan, Zhifei Liu, Jianhong Wei, Rui Xiong

**Affiliations:** Key Laboratory of Artificial Micro- and Nano-Structures of Ministry of Education and School of Physics and Technology, Wuhan University, Luojiashan Road, Wuhan 430072, China; mahongyu@whu.edu.cn (H.M.); yq.tan@whu.edu.cn (Y.T.); liuzhifei@whu.edu.cn (Z.L.)

**Keywords:** photocatalysis, CuO modification, oxygen vacancy, W_18_O_49_ microspheres, water splitting

## Abstract

Photocatalytic hydrogen evolution is a promising “green chemistry” route driven by sunlight for the direct water splitting into value-added hydrogen energy. Herein, with the object of exploring the effect of CuO loading on W_18_O_49_ photocatalytic activity, a 3D Urchin-like CuO modified W_18_O_49_ (CuO/W_18_O_49_) microspheres with different CuO loadings were synthesized via thermochemical precipitation combined with solvent-thermal method. The obtained CuO/W_18_O_49_ microspheres were analyzed by means of X-ray diffraction (XRD), scanning electron microscope (SEM), energy-dispersive X-ray spectroscopy (EDS), transmission electron microscope (TEM), X-ray photoelectron spectroscopy (XPS) and photoluminescence (PL), etc. The results infer that the urchin-like 3D morphology with a high surface area and abundant 1D nanowires promotes electron transfer, the introduction of CuO further increases the number of active sites, thereby ensuring fast interfacial charge transfer to improve photocatalytic performance. During photocatalytic H_2_ evolution from water splitting, 5 wt.% CuO/W_18_O_49_ shows the optimal performance, the H_2_ yield is almost 3.22 times that of the undoped counterparts. This work presents that oxygen-vacancy-rich heterojunction nanocomposites can be used as a new strategy to design materials with high photocatalytic activity.

## 1. Introduction

With the increasing concerns on energy and environmental problems caused by the limited fossil fuels in the world, the development of green and sustainable energy has become a major research issue nowadays. Using eco-friendly and inexhaustible solar energy to replace fossil fuels, semiconductor photocatalysis has attracted growing attention and provides a promising solution to solve these problems because of its potential application in water splitting, carbon dioxide (CO_2_) reduction, inactivating viruses and/or organic pollutants degradation, etc. [1,2,3].

Tungsten oxide (WO_3_) with a band gap of 2.5–2.8 eV is considered as an attractive n-type visible light active photocatalyst [4,5]. In addition, it’s also well known for its non-stoichiometric properties, as its lattice can withstand a considerable number of oxygen vacancies, and many secondary oxides (WO*_x_*_<3_) such as WO_2.9_, WO_2.83_, WO_2.72_ etc. have a stable crystal phase that is different from surface-reduced WO_3_. Among all the tungsten sub-oxides, monoclinic WO_2.72_, also expressed as W_18_O_49_, has received considerable attention due to its distinctive oxygen defect structure and strong light absorption up to the near-infrared region [6,7]. The presence of oxygen vacancy is conducive to the adsorption of surface species (such as CO_2_, H_2_, and NO_2_), making it a promising photocatalyst and electrode material [8]. Therefore, W_18_O_49_ is often used for solar or electric-driven water splitting, carbon dioxide fixation and rechargeable metal-air batteries, etc. However, just like other widely used photocatalysts, W_18_O_49_ also suffers from the problems of slow charge transfer and rapid electron-hole recombination in the semiconductor and electrolyte interfaces, which hinder its application in various energy processes.

Much effort has been made to improve the photocatalytic performance of W_18_O_49_ materials, such as doping, morphology control, porous-structure construction, heterojunction structure, and co-catalyst loading, etc. [9,10,11,12]. In terms of heterogeneous structure construction, Cu_2_O or CuO was found to be a class of p-type narrow-bandgap semiconductor with high activity, high selectivity and low cost cocatalysts, what is more, both are easy to compound with n-type semiconductor to form p-n junction, which is beneficial to reduce the recombination of carriers and extend their life span [13,14,15,16].

In the present work, the 3D urchin-like CuO doped W_18_O_49_ nanorod clusters (CuO/W_18_O_49_) were successfully synthesized via thermochemical precipitation combined with solvent-thermal method. The synergistic effect of CuO species and oxygen vacancies not only broadened the light absorption range of CuO/W_18_O_49_ heterojunction photocatalyst, but also enhanced the separation and transfer efficiency of carriers, resulting in high photocatalytic activity. A possible enhancement mechanism of the photocatalytic H_2_ evolution from water splitting under visible light irradiation were carefully investigated.

## 2. Experimental

### 2.1. Chemicals

All chemicals were purchased from Aladdin Chemical (Shanghai, China) and were used without further treatment.

### 2.2. Synthesis of CuO Nanoparticles (NP)

**Scheme 1:**
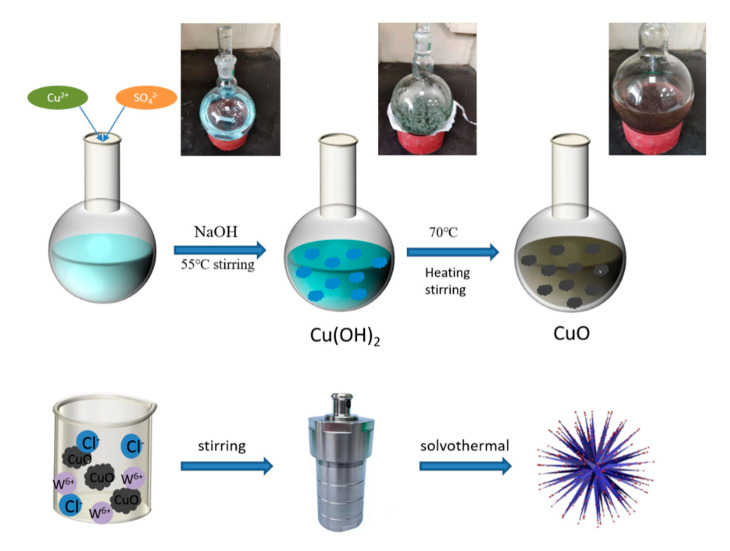
Schematic illustration of the fabrication of the CuO/W_18_O_49_ microspheres.

The CuO nanoparticles were synthesized by the thermal chemical precipitation. In a typical synthesis process, 3 mmol CuSO_4_·5H_2_O was added into 300 mL deionized water, followed a strong stirring at 55 °C until forms a blue solution. Then 7.5 mmol NaOH was added to the above solution, and the temperature was risen to 70 °C, under stirred continuously at this temperature for 2 h. After that, the precipitation was collected by centrifugation and ultrasonically cleaned with absolute ethanol (50 mL) and deionized water (50 mL, conductivity: 0.05 µS/cm) several times. Finally the product was dried at 60 °C for 6 h and further grinded to obtain a fine powder (Scheme 1).

### 2.3. Preparation of CuO/W_18_O_49_ Microspheres

The CuO/W_18_O_49_ microspheres were synthesized via a facile solvothermal method. Typically, 0.397 g of WCl_6_ was dissolved in 40 mL of absolute ethanol with intense stirring until a translucent solution formed, labeled as solution A. Then a certain mass of the CuO nanoparticles was added to 40 mL of absolute ethanol and ultrasonicated all night, labeled as solution B. Subsequently, Solution A and solution B were mixed and stirred for 2 hours. The resulting solution was then loaded into a Teflon-lined autoclave and heated in an oven of 160 °C for 24 h, followed naturally cooled to room temperature. Finally, the powder sample was collected, washed three times alternately with absolute ethanol (50 mL) and deionized water (50 mL, conductivity: 0.05 µS/cm) to remove ions and possible remnants and dried at 70 °C for overnight. Here, the sample was named as CW-*X*, *X* = 1, 3, 5, and 7, respectively, which corresponding to the weight percentage of CuO in the W_18_O_49_ powders. The real ratios were estimated by EDX technology and giving in Table S1. The pure W_18_O_49_ sample was prepared by the similar procedure just without the adding of CuO nanoparticles. The possible chemical reaction equation of W_18_O_49_ is as follows:

WCl_6_ + C_2_H_5_OH → C_2_H_5_OWCl_5_ + HCl (1)

C_2_H_5_OH + C_2_H_5_OH → C_2_H_5_OC_2_H_5_ + H_2_O (2)

C_2_H_5_OWCl_5_ + H_2_O → WO_3-x_(W_18_O_49_) + C_2_H_5_OH + HCl (3)

### 2.4. Characterization 

The phase composition of the as-prepared samples were characterized by X-ray diffraction (XRD) using a Burker D8 Advance X-ray diffractometer (Bruker AXS, Germany) with a Cu Kα radiation in a 2*θ* range from 10° to 80°. The morphologies of the as-prepared samples were examined by scanning electron microscopy (SEM, Shimadzu SSX-550) and transmission electron microscopy (TEM, JEOL JEM-2010, Tokyo, Japan). High-resolution transmission electron microscopy (HRTEM) was carried out at room temperature on a Tecnai G2 F20 field emission transmission electron microscopy (JEOL JEM-2010 FEF, Tokyo, Japan) at the accelerating voltage of 200 KV. The Energy dispersive X-ray (EDS) spectroscopy and Element mapping of CuO/W_18_O_49_ microspheres were also investigated during the FE-SEM measurement. The surface compositions and the valence band spectra of the samples were analyzed using X-ray photoelectron spectroscopy (XPS, Thermo Escalab 250 New York, NY, USA, with a monochromatic Al Kα X-ray source). All binding energies were referenced to the C 1s peak (284.6 eV) arising from adventitious carbon. The VBM values along with the estimated Eg were used to determine the conduction and valence band edges positions of the obtained samples. The UV-vis diffuse reflectance spectra (DRS) were analyzed by a U-4100 solid spectrophotometer (Shimadzu, Japan) with the wavelength range of 200–800 nm. Photoluminescence (PL) spectra of all the samples were recorded at room temperature using a Hitachi F-4600 fluorescence spectrometer (PerkinElmer, Waltham, MA, USA) with excitation at 404 nm. Electron spin resonance (ESR) spectroscopy measurements were performed at 110 K with a FA200 spectrometer (JEOL, Tokyo, Japan). 

### 2.5. Photoelectric Performance Measurement

Photocurrents measurement were performed on an electrochemical workstation (CHI660A, CH Instruments Co., Shanghai, China) in a standard three-electrode system. In a typical procedure, 10 mg of the sample was dissolved in absolute ethanol, and ultrasonic dispersion for 30 min, and then evenly spin coated on the ITO glass (1 cm × 2 cm) on the homogenizer. A sample film located in the ITO glass with the area of around 0.5 cm^2^ as a working electrode, a platinum wire and a saturated calomel electrode were used as counter and reference electrode, respectively. During the experiment, N_2_ was continuously purged. A 350 W xenon lamp equipped with a UV cutoff filter (>420 nm) was served as the visible-light source. 1 M Na_2_SO_4_ aqueous solution was used as the electrolyte in the measurement. The Mott-Schottky plot measurement was performed in the same three-electrode configuration cell setup as described above, under a constant frequency of 1 kHz and DC potential polarization in the dark.

### 2.6. Photocatalytic H_2_ Evolution Testing

The photocatalytic H_2_ generation experiments were operated in a glass gas-closed-circulation system under irradiation with a 500 W xenon lamp (CEL-HXBF 500, Beijing Perfectlight Co. Ltd., Beijing, China). In a typical testing procedure, 100 mg catalyst was ultrasonically dispersed into 50 mL (containing 15 vol% triethanolamine (TEA)) aqueous. After degassing treatment of the reaction system, the aqueous suspension was irradiated by the xenon lamp with a cut-off filter. The reaction system was stirred constantly during the light irradiation process, and the amount of H_2_ was detected by the gas chromatograph (GC2060, TCD detector and Ar carrier, Beijing Perfectlight Co. Ltd., Beijing, China).

## 3. Results and Discussion

**Figure 1:**
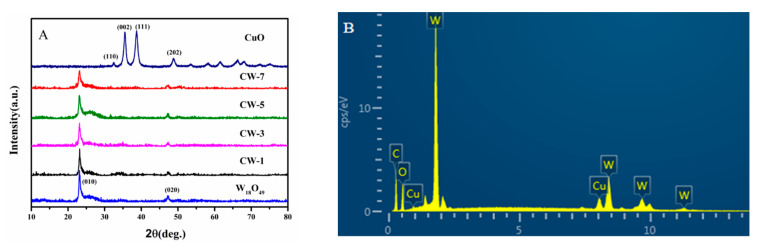
(**A**) XRD patterns of W_18_O_49,_ CuO and CuO/W_18_O_49_ samples with different contents of CuO; (**B**) the EDX spectrum of CW-5.

The XRD patterns of as-prepared samples are shown in Figure 1A. As indicated, the diffraction peak of CuO a can be indexed to the monoclinic phase of CuO and curve W_18_O_49_ can be indexed to the monoclinic W_18_O_49_ structure according to the standard diffraction peaks of JCPDS card no. 36–1451 and JCPDS card no. 71–2450. In curve W_18_O_49_, the strength of the (010) crystal plane was much higher than that of the other XRD peaks, indicating the preferential orientation of the W_18_O_49_ series samples in the growth process [17,18]. It’s found that the peaks of the as-prepared CuO/W_18_O_49_ composite samples with different CuO content only corresponded to W_18_O_49_, no peak associated with Cu, Cu_2_O, or CuO was observed, which may be due to the low content and high dispersion degree of CuO in the composite. However, the EDX spectrum of CW-5 samples (Figure 1B) only indicate the signal of W, O, C and Cu, suggesting that the main body of the sample is mainly composed of W, O and Cu elements.

**Figure 2:**
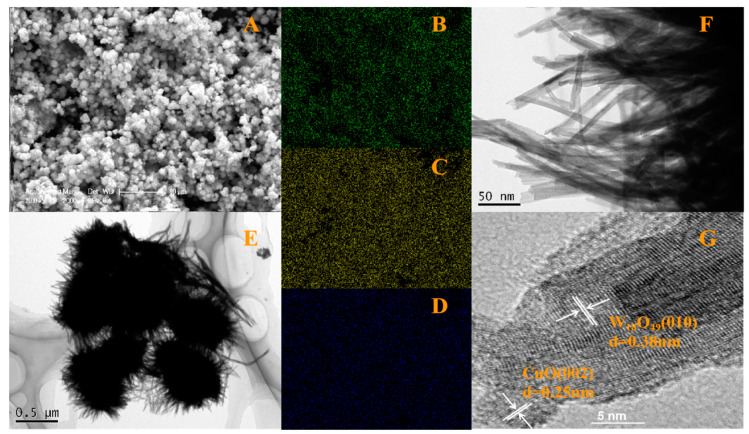
(**A**) SEM images of CuO/W_18_O_49_ microspheres, and (**B**–**D**) corresponding elemental mapping images of the elements W, O and Cu in the doped samples. (**E**) TEM image of CuO/W_18_O_49_ microspheres; (**F**) a magnified TEM image of CuO/W_18_O_49_ microspheres; (**G**) a HRTEM image of CuO/W_18_O_49_ microspheres.

The morphology of the as-prepared samples were investigated by SEM and TEM, as shown in Figure 2 and Figure S1. As seen from Figure 2A,E, the diameter of CuO/W_18_O_49_ sample is about 500–800 nm. The W, O and Cu elements mapping images in the sample are evenly distributed and overlapped (Figure 2B–D), and the results are in good agreement with the EDX analysis results. At the same time, the real content of CuO nanoparticles in CuO/W_18_O_49_ microspheres is shown in Table S1. The image in Figure S1A shows the CuO nanoparticles, which have a uniform size of about 50 nm. In Figure S1B, it can be clearly seen that the urchin-like W_18_O_49_ is composed of many nanorods, which are hundreds of nanometers in size. As shown in Figure S1C, CuO nanoparticles were successfully loaded on the surface of W_18_O_49_ microspheres. From Figure 2E,F, we can see that the CuO/W_18_O_49_ sample possesses both nano- and microscale flower-like 3D structure. The HRTEM image (Figure 2G) obtained from a random nanorod on the CuO/W_18_O_49_ microsphere shows clear interplanar spacings of approximately 0.38 nm corresponding to the (010) planes of W_18_O_49_ and 0.25 nm corresponding to the (002) planes of CuO [19,20,21,22]. In general, the 3D structure has higher specific surface area, faster carrier transport and other advantages than the corresponding 1D and 2D nanostructures and is expected to exhibit better photocatalytic activity. 

**Figure 3:**
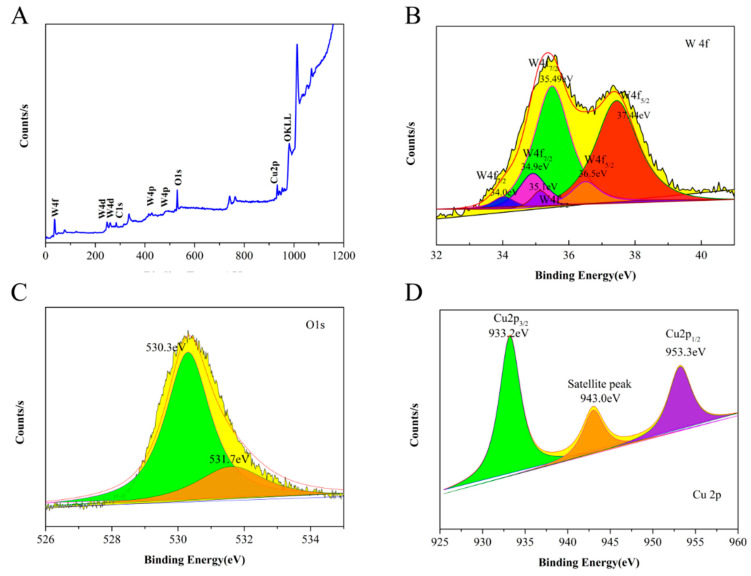
The survey spectra (**A**), W4f (**B**), O1s (**C**) and Cu2p (**D**) core-level spectrum of CW-5 sample.

To explore the surface composition and valence of elements in CW-5 samples, XPS measurement were preformed, as shown in Figure 3. The XPS full spectrum (Figure 3A) confirmed the presence of W, O, Cu and C elements in the sample and the existence of C is due to the impurities and residual organic molecules detected by XPS. The XPS spectra in Figure 3B shows the binding energy values of W 4f7/2 and W 4f5/2 at 35.49 eV and 37.44 eV ascribed to W^6+^. The two strong peaks located at 34.9 eV and 36.5 eV corresponded to the W 4f7/2 and W 4f5/2 binding energies of W^5+^. In addition, two small peaks located at 34.0 eV (W 4f7/2) and 35.1 eV (W 4f5/2) were attributed to the W^4+^. In Figure 3C, the two strong peaks located at 530.3ev and 531.7ev correspond to O^2-^ and H_2_O adsorbed on the sample surface, respectively. The XPS core-level spectra of the Cu2p recorded on CW-5 was shown in Figure 3D. As indicated, the Cu2p binding energies of CuO/W_18_O_49_ located at 933.2 eV and 953.3eV ascribed to Cu^2+^ [23,24], indicating that CuO had been successfully introduced into the W_18_O_49_ microspheres.

**Figure 4:**
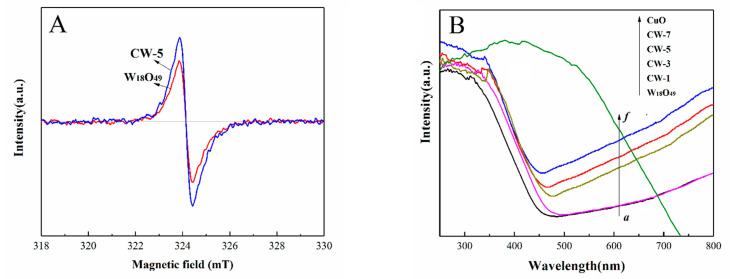

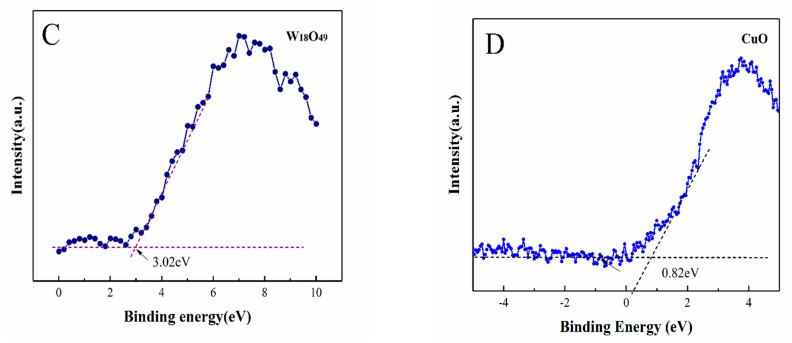
(**A**) ESR spectra of W_18_O_49_ and CuO/W_18_O_49_ samples; (**B**) Ultraviolet–visible diffuse reflectance spectra of W_18_O_49_ and CuO/W_18_O_49_ samples. (**C**) XPS VB spectra of W_18_O_49_ sample; (**D**) XPS VB spectra of CuO sample.

Electron paramagnetic resonance (EPR) spectroscopy, a high sensitivity method for unpaired spin detection, was employed to further investigate the difference of surface oxygen vacancies in our synthesized samples. As shown in Figure 4A, a rather weak oxygen vacancies signal appeared at *g* = 2.002 for W_18_O_49_ samples, while, a stronger oxygen vacancies signal can be observed in CW-5 sample, which indicated that more oxygen vacancies were generated in CuO/W_18_O_49_ composite. The potential advantage of these oxygen vacancies is that they can provide more capture sites for photogenic carriers and correspondingly prevent the rapid recombination of these carriers, thus promoting electron transfer and enhancing photocatalytic activity.

The optical absorption properties of the as-synthesized photocatalysts were investigated by UV–vis diffuse reflectance spectra (DRS). As shown in Figure 4B, the undoped W_18_O_49_ sample possesses light absorption from ultraviolet to visible light, and the absorption threshold is located at about 460 nm, corresponding to the band gap of about 2.67 eV. Due to the large number of oxygen vacancies, it exhibits a large absorption tail throughout the whole visible region. For CuO/W_18_O_49_ samples (curve b to e), the absorption edges of all samples exhibited a red shift after introduction of CuO, the amount of red-shifting increased with the increasing of CuO content in the composite, indicating that the introduction of CuO helps absorb more visible light and expands its light absorption range compared with the undoped W_18_O_49_ samples. According to the equation $Eg\approx \frac{1240}{\lambda }$, the bandgaps from W_18_O_49_ to CW-7 were changed from 2.67 eV to 2.50eV respectively. The decrease of bandgap is beneficial to widen the absorption range of visible-light, correspondingly lead to the enhancement of photocatalytic activity in visible-light region. For CuO, its bandgap is estimated to be 1.61 eV. The valence band positions of CuO and W_18_O_49_ are calculated to be 0.82 eV and 3.02 eV, respectively, according to the XPS VB measurement (Figure 4C,D). 

**Figure 5:**
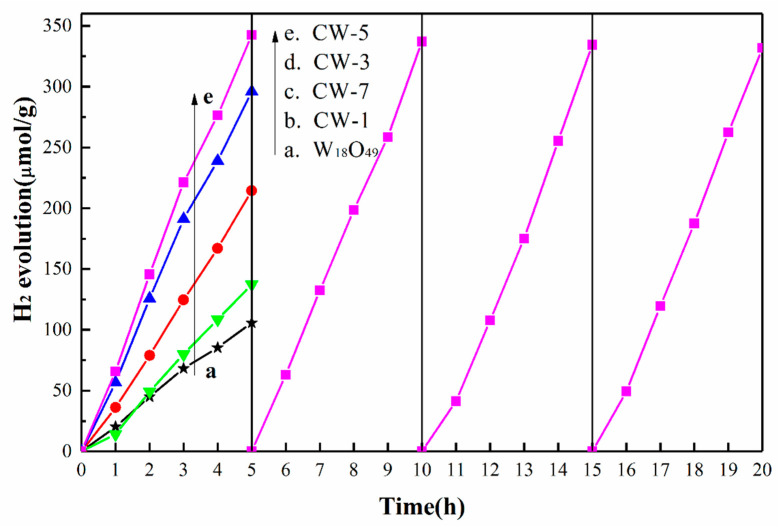
Photocatalytic H_2_ evolution amount over W_18_O_49_, and CuO/W_18_O_49_ powers as a function of irradiation time.

Hydrogen evolution from water splitting was used to evaluate photocatalytic activity of the as-prepared W_18_O_49_ and CuO/W_18_O_49_ samples. The experiments were carried out in a gas-closed circulation system under vacuum and visible light irradiation, as shown in Figure 5. The controlled experiments showed that no H_2_ was detected during the reaction in the dark. Therefore, the formation of H_2_ mainly came from the photocatalysis splitting of water. From Figure 5, it can be seen that the average H_2_ evolution rate is about 21.2 µmol/g/h after 5 h irradiation for W_18_O_49_ sample. After the introduction of CuO nanoparticles, the H_2_ evolution rate first increased with the increase of the CuO content, and then decreased with the increase of CuO content. The optimum photocatalytic activity was found in the CW-5 sample. An maximum H_2_ evolution rate (~68.4 µmol/g/h) can be observed in it, which was approximately 3.22 times that of the undoped W_18_O_49_ sample. The possible reasons are as following: on the one hand, increasing the percentage of CuO in the W_18_O_49_ samples is beneficial to the transformation of photocarriers, on the other hand, the presence of CuO will shield part of active sites, which is not conducive to the light absorption. As a balance of these two factors, the CW-5 samples exhibited the optimized photocatalytic activity. At the same time, the photocatalytic stability of the CW-5 sample was tested by illumination for 20 h. It could be seen that the hydrogen production of the sample did not decrease significantly after 4 cycles, indicating that the sample had high photocatalytic stability. 

**Figure 6:**
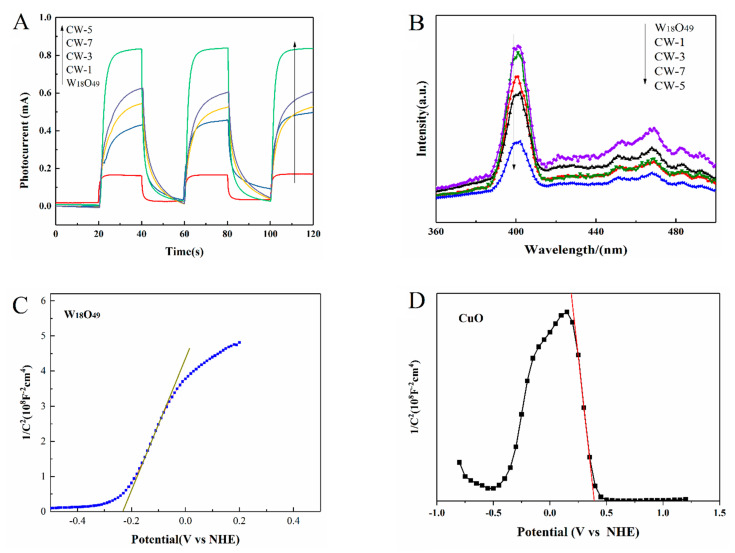
(**A**) Photocurrent generation on the catalyst electrodes coated with W_18_O_49_ or CuO/W_18_O_49_ samples; (**B**) Photoluminescence spectra of different samples; (**C**) the Mott-Schottky diagrams for W_18_O_49_ sample_;_ (**D**) the Mott-Schottky diagrams for CuO sample.

In order to further understand the role of CuO introduction, the transient photocurrent (I)—time (t) response curve of different samples with several on-off cycles under intermittent visible light irradiation was exhibited in Figure 6A. In general, the initial anodic photocurrent spike caused by electron-hole pair separation and photocurrent decay indicates that a charge recombination process is taking place [25,26,27,28]. As indicated, the photocurrent was relatively stable for several times on-off illumination, indicating that these samples had superior photoelectron-hole pair separation efficiency and long-term light stability. Besides, it can be seen that with the increase of the amount of CuO nanoparticles in W_18_O_49_ network, the photocurrent first increases and then gradually decreases. Among them, CW-5 exhibited highest peak value photocurrent density (0.83 mA), which was approximately 4.2 times that of the undoped W_18_O_49_ sample and 1.8 times that of the CW-1 sample. The results confirm that moderate introduction of CuO nanoparticles can effectively inhibit photoinduced carrier recombination because of the formation of CuO/W_18_O_49_ p-n heterojunction. 

To further investigated the separation efficiency of photogenerated carriers, the PL measurement was carried out as shown in Figure 6B. It can be seen that the pure W_18_O_49_ semiconductor exhibits a rather strong emission spectrum, indicating that the photoinduced carriers are easily recombination after being excited. After introduction CuO nanoparticle into W_18_O_49_ networks, the emission spectrum intensity first decreases gradually and then reaches its lowest value at 5wt.% CuO content in composites. Since then, the emission spectrum intensity gradually increases with the further increase of CuO. Obviously, the addition of CuO has an important influence on the photoexcited carrier’s separation efficiency of CuO/W_18_O_49_ composites. The emission intensity of the CW-5 sample is the weakest, indicating it has the highest carrier separation efficiency. This result is consistent with the I-t measurement. 

Figure 6C,D shows the Mott-Schottky diagrams for W_18_O_49_ and CuO samples, respectively. A positive slope demonstrates that W_18_O_49_ is an n-type semiconductor (vs. normal hydrogen electrode, NHE), and a negative slope indicates that CuO is a p-type semiconductor. The result infers that a p-n junction is formed between the interfaces of CuO and W_18_O_49_ samples. In general, p-type semiconductor contacts with n-type semiconductor, the p-n junction is a favor to form with intermediate electric field. Because of the difference of Fermi level, the majority carrier electrons of the W_18_O_49_ (n region) will diffuse toward the p region so that one side of the p-type CuO accumulates electrons and is negatively charged, while majority carrier holes of the CuO (p region) will diffuse toward the n region so that one side of the n-type W_18_O_49_ accumulates holes and is positive charged, forming an intermediate electric field with direction from W_18_O_49_ to CuO. Once exposed to visible light, by the intermediate electric field, the photogenerated electrons from CuO would transfer to the CB of W_18_O_49_, while the photogenerated holes would transfer from the VB of W_18_O_49_ to the VB of CuO. If so, considering the low CB level of W_18_O_49_, the electrons transferred from the CuO conduction band to the W_18_O_49_ conduction band are incapable to participate in water reduction reactions thermodynamically. As a result, p-n junction theory is contradicted with our experiment result of hydrogen production.

**Scheme 2:**
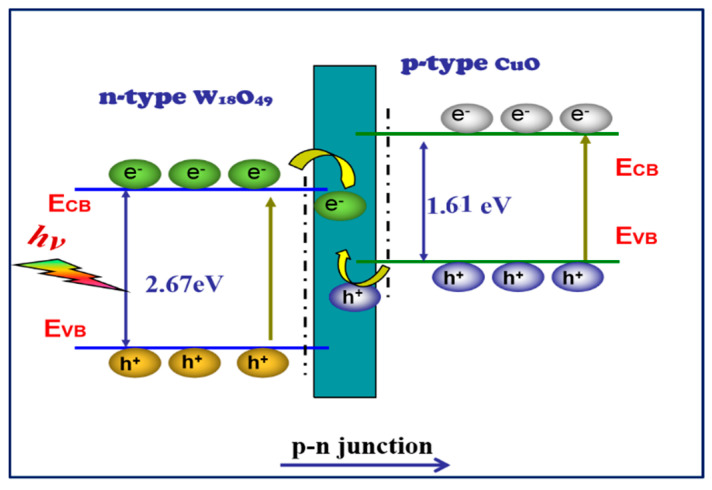
Proposed schematic diagram for the photocatalytic H_2_ evolution mechanism for the CuO/W_18_O_49_ microspheres.

Based on the above analysis, we think a direct Z-scheme photocatalytic reaction mechanism was more suitable for CuO/W_18_O_49_ heterojunction photocatalyst, as shown in Scheme 2. Here, CuO is an p-type semiconductor with bandgap of 1.61 eV and VB position of 0.82 eV; while W_18_O_49_ is an n-type semiconductor with bandgap of 2.67 eV and VB position of 3.02 eV. When the CuO/W_18_O_49_ is radiated by visible light with the photon energy higher or equal to the band gap of p-type and n-type semiconductors, the photo-induced electrons on the conduction band of W_18_O_49_ would shift to interface of CuO and W_18_O_49_, combine with the photogenerated hole from CuO. This process efficiently promotes the separation of electron-hole pairs. In addition, the electrons remaining on the CuO valence band and the holes remaining on the W_18_O_49_ conduction band have higher reduction or oxidation potentials, respectively. Because of the negative E_CB_ of CuO, these electrons exhibit strong reducibility, thereby resulting greatly enhanced photocatalytic performance.

## 4. Conclusions

In this work, a 3D Urchin-like CuO modified W_18_O_49_ (CuO/W_18_O_49_) composites were successfully synthesized via thermochemical precipitation combined with solvent-thermal method. Because of synergistic effect of CuO species and oxygen vacancies. The CuO/W_18_O_49_ composites exhibited improved light absorption ability and exposed rich catalytically reactive sites. At the same time, the Z-type mechanism of carrier behavior at the contact interface between CuO and W_18_O_49_ greatly promoted the interface charge transfer rate and carrier separation efficiency, thereby causing more photogenerated electrons and holes to participate in the reaction, corresponding resulting in a remarkable enhanced photocatalytic water splitting performance. This work demonstrates a high potential for the construction of efficient photocatalysts under visible light.

## Data Availability

Data is contained within the article or supplementary material.

## Supplementary Materials

The following are available online at https://www.mdpi.com/2079-4991/11/1/104/s1, Figure S1: The SEM images of (A) CuO, (B)W_18_O_49_ and (C) CuO/W_18_O_49_, Table S1: EDX analysis of CuO content in samples.

## References

[1] Li R.G., Weng Y.X., Zhou X., Wang X.L., Mi Y., Chong R.F., Han H.X., Li C. (2015). Achieving overall water splitting using titanium dioxide-based photocatalysts of different phases. Energy Environ. Sci..

[2] Han H.X., Li C. (2015). Photocatalysis in solar fuel production. Photocatalysis in solar fuel production. Natl. Sci. Rev..

[3] Hsu M.H., Chang C.J., Weng H.T. (2016). Efficient H2 production using Ag2S-coupled ZnO@ZnS core-shell nanorods decorated metal wire mesh as an immobilized hierarchical photocatalyst. ACS Sustain. Chem. Eng..

[4] Li Y.S., Tang Z.L., Zhang J.Y., Zhang Z.T. (2017). Fabrication of vertical orthorhombic/hexagonal tungsten oxide phase junction with high photocatalytic performance. Appl. Catal. B Environ..

[5] Singh T., Müller R., Singh J., Mathur S. (2015). Tailoring surface states in WO3 photoanodes for efficient photoelectrochemical water splitting. Appl. Surf. Sci..

[6] Valentin D., Pacchioni G. (2014). Spectroscopic properties of doped and defective semiconducting oxides from hybrid density functional calculations. Tungsten oxides for photocatalysis, electrochemistry, and phototherapy. Acc. Chem. Res..

[7] Huang Z.F., Song J.J., Pan L., Zhang X.W., Wang L., Zou J.J. (2015). Tungsten oxides for photocatalysis, electrochemistry, and phototherapy. Adv. Mater..

[8] Yan J., Wang T., Wu G., Dai W., Guan N., Li L., Gong J. (2015). Tungsten oxide single crystal nanosheets for enhanced multichannel solar light harvesting. Adv. Mater..

[9] Pan L., Zhang J.W., Jia X., Ma Y.H., Zhang X.W., Wang L., Zou J.J. (2017). Highly efficient Z-scheme WO3–x quantum dots/TiO2 for photocatalytic hydrogen generation. Chin. J. Catal..

[10] Xi G.C., Ye J.H., Ma Q., Su N., Bai H., Wang C. (2012). In situ growth of metal particles on 3D urchin-like WO3 nanostructures. J. Am. Chem. Soc..

[11] Sun Y., Wang W., Qin J.W., Zhao D., Mao B.G., Xiao Y., Cao M.H. (2016). Oxygen vacancy-rich mesoporous W18O49 nanobelts with ultrahigh initial Coulombic efficiency toward high-performance lithium storage. Electrochim. Acta.

[12] Zhong X., Sun Y.Y., Chen X.L., Zhuang G.L., Li X.N., Wang J.G. (2016). Mo doping induced more active sites in urchin-like W18O49 nanostructure with remarkably enhanced performance for hydrogen evolution reaction. Adv. Funct. Mater..

[13] Reddy N.L., Kumar S., Krishnan V., Sathish M., Shankar M.V. (2017). Multifunctional Cu/Ag quantum dots on TiO2 nanotubes as highly efficient photocatalysts for enhanced solar hydrogen evolution. J. Catal..

[14] Li W., He S.O., Su Z.Y., Xu W., Wang X.C. (2019). A BiOCl-CuO photocatalyst based on p-n heterojunction and its photocatalytic performance under visible-light. Appl. Surf. Sci..

[15] Li G.J., Huang J.Q., Deng Z.H., Chen J., Huang Q.F., Liu Z.G., Guo W., Cao R. (2019). Highly active photocatalyst of CuOx modified TiO2 arrays for hydrogen generation. Cryst. Growth Des..

[16] Sun L.M., Zhuang Y., Yuan Y.S., Zhan W.W., Wang X.J., Han X.G., Zhao Y.L. (2019). Nitrogen-doped carbon-coated CuO-In2O3 p-n heterojunction for remarkable photocatalytic hydrogen evolution. Adv. Energy Mater..

[17] Zhao Y.Y., Tang Q.W., Yang P.Z., He B.L. (2017). Robust electrocatalysts from metal doped W18O49 nanofibers for hydrogen evolution. Chem. Commun..

[18] Lou Z.Z., Gu Q., Liao Y.S., Yu S.J., Xue C. (2016). Promoting Pd-catalyzed suzuki coupling reactions through near-infrared plasmon excitation of WO3-x nanowires. Appl. Catal. B Environ..

[19] Bai H., Su N., Li W., Zhang X., Yan Y., Li P., Ouyang S., Ye J., Xi G. (2013). W18O49 nanowire networks for catalyzed dehydration of isopropyl alcohol to propylene under visible light. J. Mater. Chem. A.

[20] Spadavecchia F., Ardizzone S., Cappelletti G., Cheng L.W., Ju Y.R., Payamyar P., Prim D., Rao J.Y., Will C., Koziej D. (2015). Large-area alignment of tungsten oxide nanowires over flat and patterned substrates for room-temperature gas sensing. Angew. Chem. Int. Ed..

[21] Hou H., Shang M., Gao F., Wang L., Liu Q., Zheng J., Yang Z., Yang W. (2016). Highly efficient photocatalytic hydrogen evolution in ternary hybrid TiO2/CuO/Cu thoroughly mesoporous nanofibers. ACS Appl. Mater. Interfaces.

[22] Basnet P., Anderson E., Zhao Y.P. (2019). Hybrid CuxO–TiO2 Nanopowders Prepared by Ball Milling for Solar Energy Conversion and Visible-Light-Induced Wastewater Treatment. ACS Appl. Nano Mater..

[23] Xie Y., Yin Y.L., Zeng S.H., Gao M.Y., Su H.Q. (2017). Coexistence of Cu+ and Cu2+ in star-shaped CeO2/CuxO catalyst for preferential CO oxidation. Catal. Commun..

[24] Schön G. (1973). ESCA studies of Cu, Cu2O and CuO. Surf. Sci..

[25] Lei F., Sun Y., Liu K., Gao S., Liang L., Pan B., Xie Y. (2014). Oxygen vacancies confined in ultrathin indium oxide porous sheets for promoted visible-light water splitting. J. Am. Chem. Soc..

[26] Wang G., Wang H., Ling Y., Tang Y., Yang X., Fitzmorris R.C., Wang C., Zhang J.Z., Li Y. (2011). Hydrogen-treated TiO2 nanowire arrays for photoelectrochemical water splitting. Nano Lett..

[27] Sakai N., Ebina Y., Takada K., Sasaki T. (2004). Electronic band structure of titania semiconductor nanosheets revealed by electrochemical and photoelectrochemical studies. J. Am. Chem. Soc..

[28] Falciola M., Ceotto D., Lotti J. (2013). Investigation and optimization of photocurrent transient measurements on nano-TiO2. Appl. Electrochem..

