# Supplementary material for: 3D Urchin-Like CuO Modified W18O49 Nanostructures for Promoted Photocatalytic Hydrogen Evolution under Visible Light Irradiation
Source: Nanomaterials (Basel). 2021 Jan 4;11(1):104. doi: 10.3390/nano11010104 (PMC7823848; doi:10.3390/nano11010104)
Supplement: Supplementary file 1 [file nanomaterials-11-00104-s001.pdf]

Supporting information

3D Urchin-like CuO modified $W_{18}O_{49}$ Nanostructures for Promoted Photocatalytic Hydrogen Evolution under Visible Light Irradiation

Hongyu Ma, Yaqi Tan, Zhifei Liu, Jianhong Wei*, Rui Xiong*

Key Laboratory of Artificial Micro- and Nano-structures of Ministry of Education and School of Physics and Technology, Wuhan University, Luojiashan Road, Wuhan, 430072, P. R. China

* Corresponding author:

E-mail: jhwei@whu.edu.cn; xiongrui@whu.edu.cn

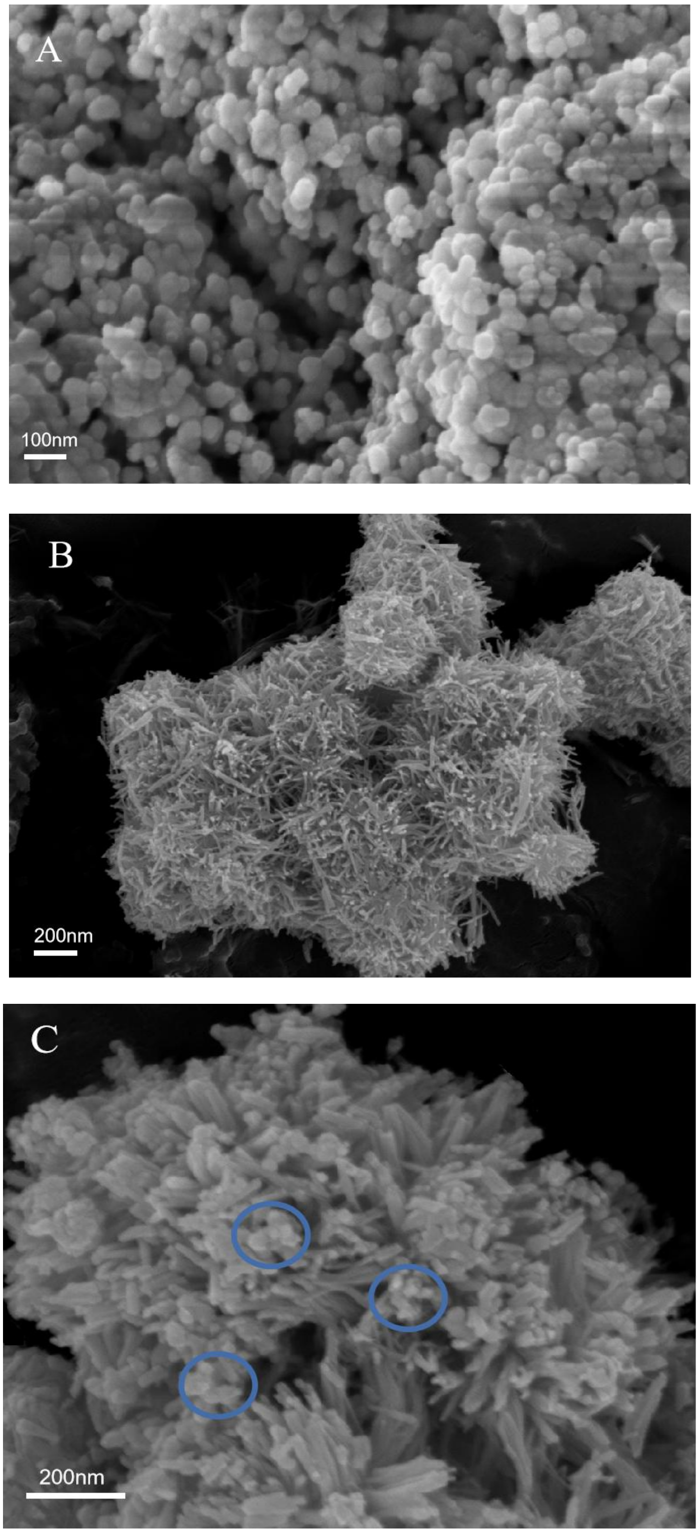

Figure S1 The SEM images of (A) CuO, (B)$W_{18}O_{49}$ and (C) CuO/$W_{18}O_{49}$。

Table 1 EDX analysis of CuO content in samples

Sample	Real content (CuO)	Theoretical content (CuO)
CW-1	0.87%	1%
CW-3	2.3%	3%
CW-5	4.6%	5%
CW-7	6.2%	7%
